# SOX-9 as a prognostic marker in gastric adenocarcinoma

**DOI:** 10.17305/bb.2024.11928

**Published:** 2025-01-27

**Authors:** Efe Yetişgin, Aysun Gökçe, Kutsal Doğan

**Affiliations:** 1 Dışkapı Yıldırım Beyazıt Training and Research Hospital Department of Pathology, Ankara, Türkiye

**Keywords:** Gastric cancer, GC, SRY-box transcription factor 9, SOX9, adenocarcinoma, prognosis, stomach

## Abstract

SRY-box transcription factor 9 (SOX9) has been reported to be overexpressed in a wide variety of gastrointestinal malignancies. While its role has been studied in gastric cancer (GC), the results remain conflicting. This study aimed to evaluate the relationship between SOX9 immunohistochemistry results and the pathological and clinical characteristics of gastric adenocarcinoma, assessing its potential as a prognostic marker. Gastric tissue samples from 150 patients with gastric cancer were included in the study. Tissue sections were stained using an anti-SOX9 antibody, and relevant data were retrospectively collected from digital records. Immunostaining results were scored based on the proportion and intensity of stained nuclei throughout the tumor. A final immunostaining score was calculated by multiplying the SOX9 intensity score by the proportion score. Strong SOX9 nuclear staining was observed in 68 patients (45.3%), while moderate staining was seen in 60 patients (40%). SOX9 nuclear staining was absent in three patients (2%). A final SOX9 immunostaining score of ≥10, classified as high expression, was identified in 60 patients (40%). Patients with higher SOX9 expression or strong intensity scores exhibited significantly larger tumor sizes, higher rates of perineural and vascular invasion, more advanced Tumor (T) or lymph node staging, and greater likelihoods of lymphatic or distant metastases compared to those with lower SOX9 expression or intensity scores (all *P* < 0.05). These findings suggest that SOX9 staining intensity and expression are associated with increased tumor malignancy and disease progression. Therefore, SOX9 may serve as a prognostic pathological indicator in GC patients.

## Introduction

Gastric cancer (GC) is diagnosed in over one million individuals annually [1]. Despite advancements in management, the prognosis remains poor [1, 2]. The primary challenge is that GC is often diagnosed at an inoperable stage due to its non-specific early symptoms and the presence of regional or distant metastases at diagnosis [3]. This subtle progression to advanced stages complicates staging with the widely-used tumor-node-metastasis (TNM) system, as patients at the same TNM stage often exhibit varying outcomes [4]. In the absence of significant diagnostic or prognostic breakthroughs, improving the sensitivity of TNM staging remains a critical goal. This can be achieved through the identification of new prognostic indicators [5].

Advancements in molecular biology have identified several biomarkers that enhance prognostic accuracy and therapeutic strategies for GC. For example, anti-ERBB2 therapy is effective in patients with unresectable or metastatic/recurrent ERBB2-positive GC, with *ERBB2* testing serving as a predictor of therapeutic response [6]. ERBB2 status is primarily determined via immunohistochemistry, while in situ hybridization is recommended for equivocal cases. EGFR amplification has been identified as an independent prognostic factor in stage II/III GC [7]. Similarly, c-MET status has been proposed as an independent prognostic indicator for patients with unresectable or recurrent GC undergoing standard chemotherapy [8]. Moreover, GC cases associated with Epstein–Barr virus (EBV) positivity or microsatellite instability (MSI)/mismatch repair (*MMR*) deficiency tend to have better prognoses compared to EBV-negative or microsatellite-stable (MSS)/*MMR-*proficient cases [9, 10]. PD-1/PD-L1 expression has been found to be significantly elevated in GC subtypes characterized by MSI and EBV positivity, making these subtypes promising candidates for immunotherapy targeting the PD-1/PD-L1 pathway [9].

Sex-determining region Y-related high mobility group box 9 (SOX9) is a key regulator in various stages of embryonic development, including sex determination, neurogenesis, neural crest development, and chondrogenesis [11]. In the gastrointestinal system, SOX9 is expressed in the nuclei of crypt cells from the embryonic stage and plays a role in endoderm differentiation and intestinal epithelial homeostasis via the Wnt/β-catenin signaling pathway [12]. Over the past two decades, altered SOX9 expression has been studied in various cancers—including colorectal, lung, laryngeal, esophageal, hepatocellular, and breast cancers—and has been associated with advanced TNM stages, higher tumor grades, lymphovascular invasion, and distant metastases [13]. Studies investigating SOX9 in GC are relatively recent, and the reported effect sizes for its diagnostic, prognostic, and outcome-related impacts often vary widely, resulting in extreme heterogeneity in meta-analyses [13, 14]. The literature also reveals conflicting findings, with substantial differences between in vitro (cell-line and organoid) studies and clinical data [15]. These discrepancies may arise from the diverse physiological roles of SOX9 in stem cells, progenitor cells (expansion properties), T cells, and epithelial cells [16–18]. Additionally, variations in the cellular source of SOX9 expression (cytoplasmic vs nuclear) and its site of expression within the gastrointestinal tract appear to influence its downstream effects [17, 19]. As such, the prognostic value of preoperative SOX9 evaluation in GC remains unclear, necessitating further investigation.

Our study aimed to explore the relationship between SOX9 expression and pathological data in gastric adenocarcinoma and to evaluate its potential as a prognostic immunohistochemical marker.

## Materials and methods

### Patients and samples

We collected 150 primary gastric adenocarcinoma tissue samples resected from patients who underwent primary surgical treatment between 2017 and 2022 at Ankara Dışkapı Yıldırım Beyazıt Training and Research Hospital (Health Sciences University). The inclusion criteria were as follows: histopathologically confirmed gastric adenocarcinoma, availability of complete clinical, treatment, and follow-up data, and the presence of adequate tumor tissue samples. Patients were excluded if they had gastric tumors of non-epithelial origin, metastatic tumors originating from other organs, prior neoadjuvant therapy, inadequate or unreliable clinical data, low-quality tissue specimens, or tissue blocks withinsufficient material. All research procedures were reviewed and approved by the Research Ethics Committee of Ankara Dışkapı Yıldırım Beyazıt Training and Research Hospital (Health Sciences University) (approval: 11.29.2021/125-08) and conducted in accordance with the Declaration of Helsinki.

Clinicopathological data were collected, including patient age, sex, tumor differentiation, size, histological subtype, vascular and perineural invasion, lymph node involvement, distant metastasis, and TNM staging. Histological subtypes were classified using World Health Organization (WHO) criteria. Lymph node involvement stage (pN), depth of invasion stage (pT), and total pathological staging were also determined according to the WHO classification. It is worth noting that for 36 patients whose pathology reports were prepared by our pathology department but whose treatment did not continue at our hospital, distant organ metastasis data could not be obtained.

### Immunohistochemistry

Formalin-fixed, paraffin-embedded gastric tissue specimens from 150 cases of gastric adenocarcinoma were prepared using standard pathology department procedures to facilitate immunohistochemistry analysis. Tissue microarray assays were performed on 4-µm-thick serial sections of routinely processed tumor samples. Specimens underwent deparaffinization with a graded series of ethanol dilutions, followed by rehydration. Heat-induced antigen retrieval was carried out in a 10 mM Tris-EDTA buffer (pH 9.0) for 40 min. For immunohistochemical staining, the sections were incubated overnight at 4 ^∘^C with a 1:100 dilution of a polyclonal rabbit anti-SOX9 antibody. Endogenous peroxidase activity was blocked by treating the specimens with horseradish peroxidase for 1 h at room temperature. Staining was visualized using a 3,3′-diaminobenzidine tetrahydrochloride solution for 7 min. Finally, the sections were counterstained with hematoxylin, dehydrated, and mounted.

### Evaluation of SOX9 staining

**Figure 1.**
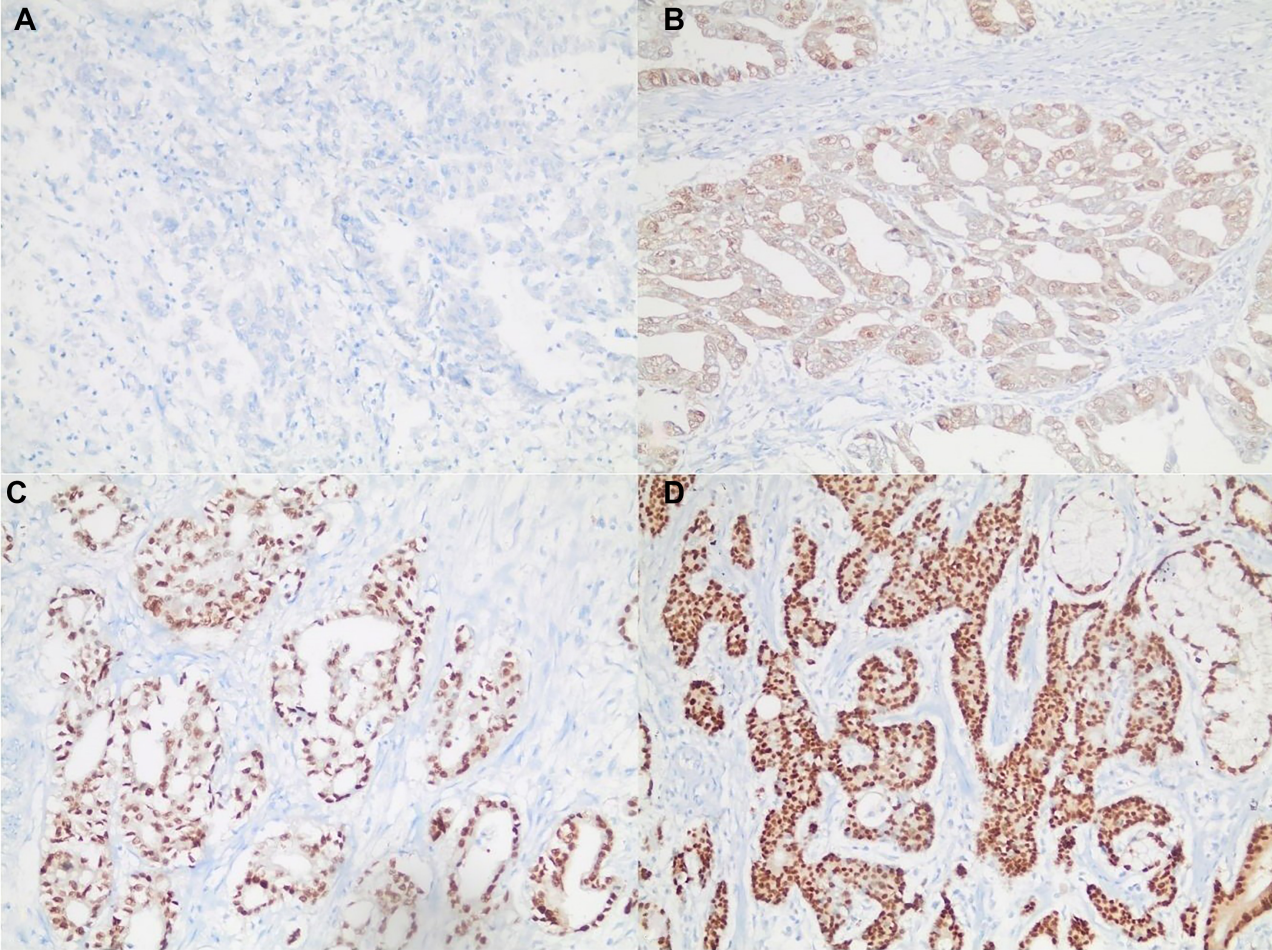
**The intensity score for SOX9 staining.** (A) Score 0 (Negative, H&E ×200); (B) Score 1 (Weak staining, H&E ×200); (C) Score 2 (Moderate staining, H&E ×200); (D) Score 3 (Strong staining, H&E ×200). SOX9: SRY-box transcription factor 9.

Immunohistochemistry analysis for SOX9 was independently evaluated by two experienced pathologists, blinded to patients’ clinicopathological outcomes. Discrepancies between evaluations were resolved through joint review and consensus. Immunostaining results were semi-quantitatively scored based on the proportion and intensity of stained nuclei throughout the tumor, with all slides scanned at ×100 magnification [20]. Artefactual staining areas were excluded from the evaluation, focusing instead on regions where tumor cells exhibited the most intense staining. The intensity score for SOX9 nuclear staining was categorized into four stages: 0 (negative, no positively stained cell nuclei), 1 (weak, yellow staining), 2 (moderate, brown staining), and 3 (strong, blackish-brown staining) (Figure 1). The proportion of tumor cell nuclei exhibiting positive reactivity with SOX9 was classified into six degrees: 0 (negative), 1 (positivity detected in ≤ 1% of the total tumor), 2 (> 1% and ≤ 10% positivity), 3 (positive cells > 10% and ≤ 33%), 4 (> 33% and ≤ 66% positivity), and 5 (positivity detected in more than 66% of the total tumor). The final immunostaining score was derived by multiplying the SOX9 intensity score with the proportion score. A score of ≥ 10 was considered indicative of high SOX9 expression, while a score of < 10 denoted low SOX9 expression.

### Statistical analysis

Data were collected using an SPSS database, and analyses were conducted in SPSS software (version 25.0; IBM, USA) with the classical significance threshold of *P* < 0.05. The normality of variable distributions was assessed through histograms and Q–Q plots. Descriptive statistics were presented as mean ± standard deviation for normally distributed continuous variables, median (minimum–maximum) for non-normally distributed continuous variables, and frequency (percentage) for categorical variables. Between-group comparisons were carried out using the independent samples *t*-test for normally distributed continuous variables, the Mann–Whitney *U* test for non-normally distributed continuous variables, and the chi-square test for categorical variables. Multivariable analyses were also conducted using binomial logistic regression and ordinal logistic regression models to assess the independent effects of variables on categorical outcomes. The regression models were adjusted for potential confounders, such as age and gender, where applicable. Results of the regression analyses were reported as estimated coefficients (β) or odds ratios (ORs) with 95% confidence intervals (CIs) and corresponding *P* values.

## Results

The mean age of patients at diagnosis was 67.4 ± 11.8 years, with 106 patients (70.7%) identified as male. Tissue specimens revealed high differentiation in 72 cases (48%), moderate differentiation in 70 cases (46.7%), and well differentiation in 8 cases (5.3%). The median tumor size was 5 cm, ranging from 1 to 15 cm. According to the WHO classification, the most prevalent subtype was tubular, observed in 85 patients (56.7%). Vascular invasion was identified in 91 patients (60.7%), while perineural invasion was present in 82 cases (54.7%). Lymph node metastasis was detected in 72.7% of the subjects. Among 114 patients examined for distant metastasis, 45 (39.5%) had confirmed cases. Based on the TNM classification, patients were distributed across various stages: 11 (7.3%) in Stage IA, 10 (6.7%) in Stage IB, 19 (12.7%) in Stage IIA, 7 (4.7%) in Stage IIB, 23 (15.3%) in Stage IIIA, 21 (14%) in Stage IIIB, 14 (9.3%) in Stage IIIC, and 45 (30%) in Stage IV.

**Figure 2.**
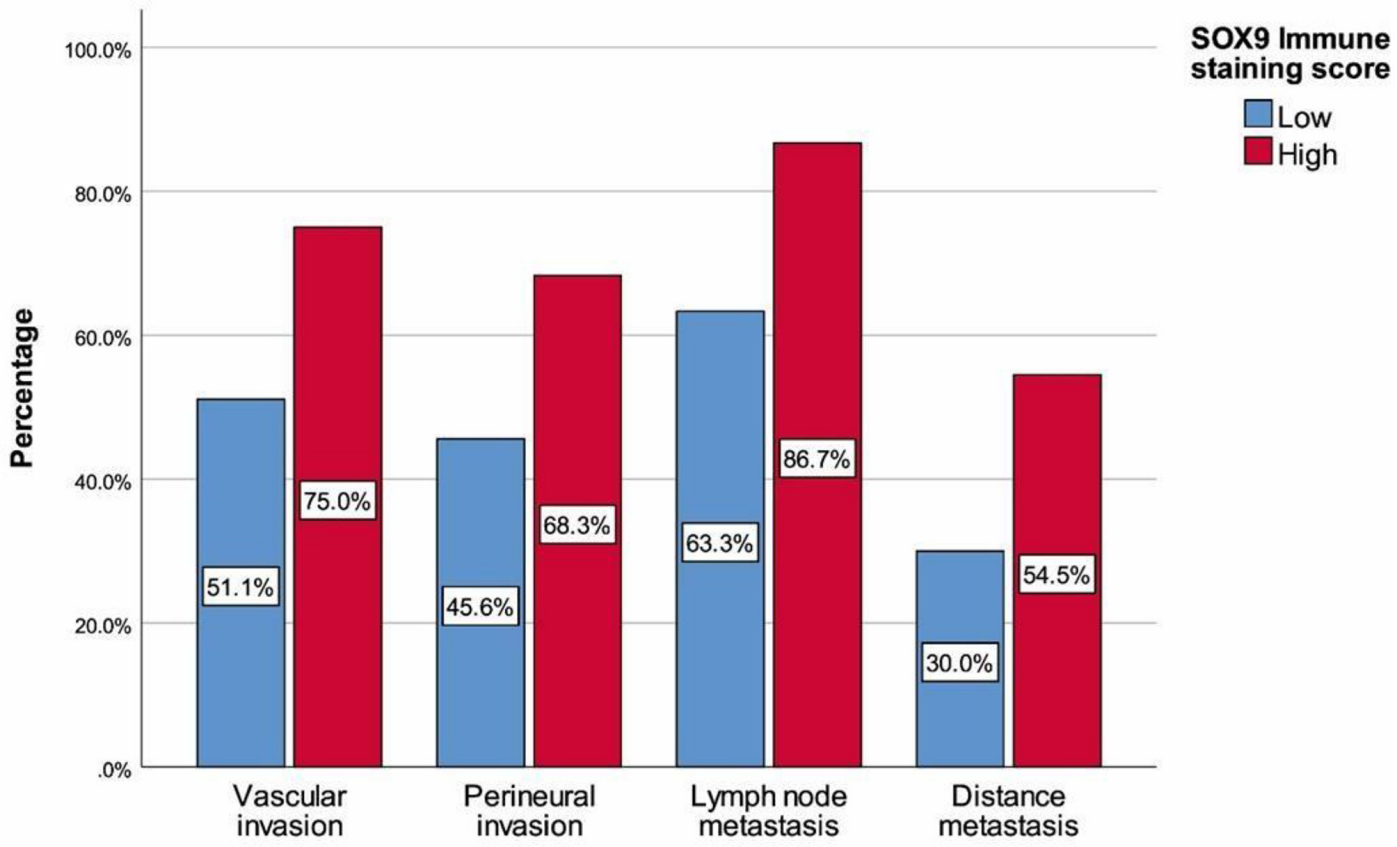
**Invasion and metastasis percentages with regard to SOX9 immune staining score.** Higher SOX9 expression is related to increased perineural and vascular invasion, and greater lymph node and distant metastases. SOX9: SRY-box transcription factor 9.

**Figure 3.**
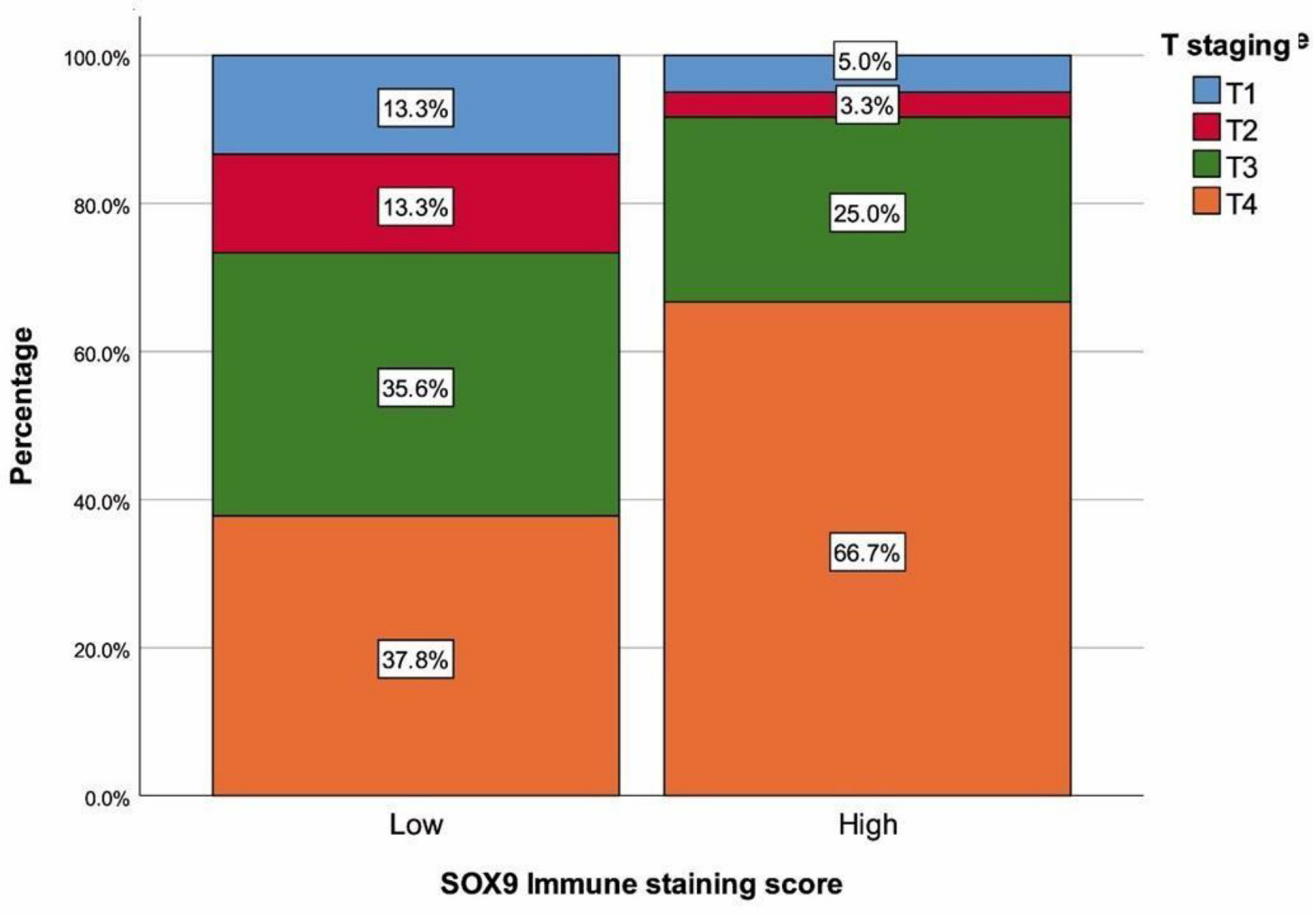
**T staging with regard to SOX9 immune staining score.** High SOX9 immunostaining scores are related to advanced T staging. SOX9: SRY-box transcription factor 9.

**Figure 4.**
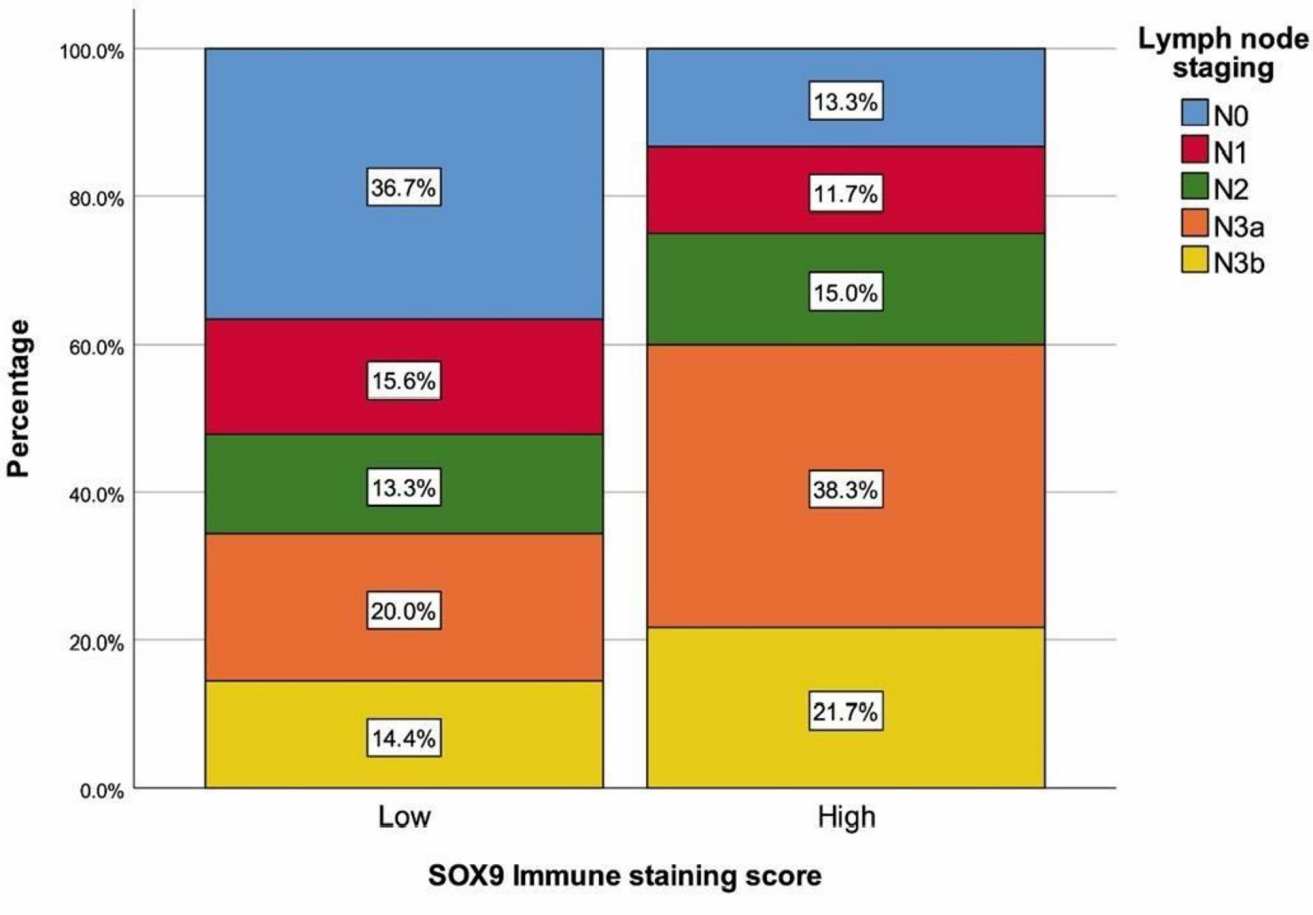
**Lymph node staging with regard to SOX9 immune staining score.** Increased SOX9 immunostaining scores are associated with advanced lymph node staging. SOX9: SRY-box transcription factor 9.

The SOX9 staining scores for patients are summarized in Table 1. Strong intensity of SOX9 nuclear staining was observed in 68 (45.3%) patients, while a moderate staining score was revealed in 60 (40%) patients. Absence of SOX9 nuclear staining was found in 3 (2%) gastric adenocarcinoma patients. Among all cases, the staining proportion score of tumor cells was stage 5 in 64 (42.6%) cases, stage 4 in 42 cases, and stage 3 in 29 cases. The final SOX9 immune staining score was ≥ 10 in 60 (40%) patients, indicating high SOX9 expression. Table 2 summarizes the demographics and tumor characteristics of patients based on the final SOX9 immune staining score. Patients with higher SOX9 expression demonstrated longer tumor sizes, higher rates of perineural and vascular invasion, and a greater presence of lymph nodes and distant metastases compared to those with lower expression (all, *P* < 0.05) (Figure 2). Patients with higher SOX9 immunostaining scores exhibited higher T and lymph node staging proportions (Figures 3 and 4). In cases with stage T4 or N3a, a significantly higher number and proportion of patients had a final SOX9 staining score (*P* ═ 0.003 and 0.011, respectively). Conversely, in those with NO or T2 stage, the number and proportion of patients with low SOX9 expression were higher than those with high expression (*P* ═ 0.011 and 0.003, respectively) (Figures 3 and 4). No differences were found between the two groups with different SOX9 expression scores in terms of age, sex, tumor differentiation or subtype, and TNM staging (all, *P* > 0.05).

**Table 1:** SOX9 scores of patients

**Intensity score**	***n* (%)**
0 (Negative)	3 (2.0%)
1 (Weak)	19 (12.7%)
2 (Moderate)	60 (40.0%)
3 (Strong)	68 (45.3%)
Proportion score (%)	60 (0–95)
0 (Negative)	3 (2.0%)
1 (≤1%)	3 (2.0%)
2 (>1%–≤10%)	9 (6.0%)
3 (>10%–≤33%)	29 (19.3%)
4 (>33%–≤66%)	42 (28.0%)
5 (>66%)	64 (42.7%)
Immune staining score	9 (0–15)
Low (≤10)	90 (60.0%)
High (>10)	60 (40.0%)

Descriptive statistics were presented by using median (minimum–maximum) for non-normally distributed continuous variables and frequency (percentage) for categorical variables. SOX9: SRY-box transcription factor 9.

**Table 2:** Demographics and tumor characteristics of patients with regard to SOX9 immune staining score

**SOX9 immune staining score**				
**Variables**	**All patients (*n* ═ 150)**	**Low, ≤10 (*n* ═ 90)**	**High, >10 (*n* ═ 60)**	***P* value**
Age	67.4 ± 11.8	68.1 ± 11.6	66.3 ± 12.2	0.368^a^
*Sex*				
Female	44 (29.3%)	26 (28.9%)	18 (30.0%)	0.884^b^
Male	106 (70.7%)	64 (71.1%)	42 (70.0%)	
*Differentiation*				
Well	8 (5.3%)	6 (6.7%)	2 (3.3%)	
Moderate	70 (46.7%)	48 (53.3%)	22 (36.7%)	0.053^b^
Poor	72 (48.0%)	36 (40.0%)	36 (60.0%)	
Tumor size, cm	5 (1–15)	4 (1–15)	6 (2–15)	0.005^c^
*Subtype*				
Tubular	85 (56.7%)	56 (62.2%)	29 (48.3%)	
Papillary	13 (8.7%)	10 (11.1%)	3 (5.0%)	0.074^b^
Mucinous	17 (11.3%)	8 (8.9%)	9 (15.0%)	
Poorly cohesive	35 (23.3%)	16 (17.8%)	19 (31.7%)	
Vascular invasion	91 (60.7%)	46 (51.1%)	45 (75.0%)	0.003^b^
Perineural invasion	82 (54.7%)	41 (45.6%)	41 (68.3%)	0.006^b^
Lymph node metastasis	109 (72.7%)	57 (63.3%)	52 (86.7%)	0.002^b^
Distance metastasis*	45 (39.5%)	21 (30.0%)	24 (54.5%)	0.009^b^
*T staging*				
T1	15 (10.0%)	12 (13.3%)	3 (5.0%)	
T2	14 (9.3%)	12 (13.3%)^+^	2 (3.3%)^+^	0.003^b^
T3	47 (31.3%)	32 (35.6%)	15 (25.0%)	
T4	74 (49.3%)	34 (37.8%)^+^	40 (66.7%)^+^	
*Lymph node staging*				
N0	41 (27.3%)	33 (36.7%)^+^	8 (13.3%)^+^	
N1	21 (14.0%)	14 (15.6%)	7 (11.7%)	
N2	21 (14.0%)	12 (13.3%)	9 (15.0%)	0.011^b^
N3a	41 (27.3%)	18 (20.0%)^+^	23 (38.3%)^+^	
N3b	26 (17.3%)	13 (14.4%)	13 (21.7%)	
*Total staging (TNM)*				
1A	11 (7.3%)	9 (10.0%)	2 (3.3%)	
1B	10 (6.7%)	8 (8.9%)	2 (3.3%)	
2A	19 (12.7%)	16 (17.8%)	3 (5.0%)	
2B	7 (4.7%)	4 (4.4%)	3 (5.0%)	0.082^b^
3A	23 (15.3%)	13 (14.4%)	10 (16.7%)	
3B	21 (14.0%)	12 (13.3%)	9 (15.0%)	
3C	14 (9.3%)	7 (7.8%)	7 (11.7%)	
4	45 (30.0%)	21 (23.3%)	24 (40.0%)	

Descriptive statistics were presented by using mean ± standard deviation for normally distributed continuous variables, median (minimum–maximum) for non-normally distributed continuous variables and frequency (percentage) for categorical variables. *: Data was available for 114 patients, +: Significantly different category, a: Independent samples *t*-test, b: Chi-square test, c: Mann–Whitney *U* test. SOX9: SRY-box transcription factor 9; TNM: Tumor-node-metastasis.

Demographics and tumor features of subjects according to SOX9 intensity score are presented in Table 3. Patients with a strong SOX9 intensity score exhibited poor or moderate differentiation, longer tumor sizes, higher rates of perineural and vascular invasion, and a greater presence of lymph nodes and distant metastases compared to those with a non-strong SOX-9 intensity score (all, *P* < 0.05). While the number and rate of patients with strong intensity scores were low in the T1 (4.4%) and T2 (2.9%) stages, they were high in the T4 stage (66.2%) (*P* ═ 0.001). The highest number and percentage of patients with a strong intensity score were in lymph node staging N3a, while those with a non-strong score were at N0 (*P* ═ <0.001). Patients with higher SOX9 intensity scores had proportionally higher T and lymph node staging. A higher proportion of patients with a strong intensity score was observed in TNM staging 4 (*P* ═ 0.008). A statistically significant relationship was observed between SOX9 staining proportion score and perineural invasion (*P* ═ 0.008). Additionally, a higher SOX9 staining proportion score was observed in patients with T4 stage (*P* ═ 0.031) (Table 4).

**Table 3:** Demographics and tumor characteristics of patients with regard to SOX9 intensity score

**SOX9 intensity score**			
**Variables**	**Negative–moderate (*n* ═ 82)**	**Strong (*n* ═ 68)**	***P* value**
Age	67.3 ± 10.7	67.4 ± 13.1	0.945^a^
*Sex*			
Female	21 (25.6%)	23 (33.8%)	0.271^b^
Male	61 (74.4%)	45 (66.2%)	
*Differentiation*			
Well	6 (7.3%)	2 (2.9%)	
Moderate	45 (54.9%)^+^	25 (36.8%)^+^	0.020^b^
Poor	31 (37.8%)^+^	41 (60.3%)^+^	
Tumor size, cm	4 (1–14)	5.5 (2–15)	0.009^c^
*Subtype*			
Tubular	50 (61.0%)	35 (51.5%)	
Papillary	10 (12.2%)	3 (4.4%)	0.085^b^
Mucinous	8 (9.8%)	9 (13.2%)	
Poorly cohesive	14 (17.1%)	21 (30.9%)	
Vascular invasion	41 (50.0%)	50 (73.5%)	0.003^b^
Perineural invasion	38 (46.3%)	44 (64.7%)	0.025^b^
Lymph node metastasis	49 (59.8%)	60 (88.2%)	<0.001^b^
Distance metastasis*	18 (28.6%)	27 (52.9%)	0.008^b^
*T staging*			
T1	12 (14.6%)^+^	3 (4.4%)^+^	
T2	12 (14.6%)^+^	2 (2.9%)^+^	0.001^b^
T3	29 (35.4%)	18 (26.5%)	
T4	29 (35.4%)^+^	45 (66.2%)^+^	
*Lymph node staging*			
N0	33 (40.2%)^+^	8 (11.8%)^+^	
N1	14 (17.1%)	7 (10.3%)	
N2	10 (12.2%)	11 (16.2%)	<0.001^b^
N3a	15 (18.3%)^+^	26 (38.2%)^+^	
N3b	10 (12.2%)	16 (23.5%)	
*Total staging (TNM)*			
1A	9 (11.0%)	2 (2.9%)	
1B	8 (9.8%)	2 (2.9%)	
2A	16 (19.5%)^+^	3 (4.4%)^+^	
2B	4 (4.9%)	3 (4.4%)	0.008^b^
3A	11 (13.4%)	12 (17.6%)	
3B	11 (13.4%)	10 (14.7%)	
3C	5 (6.1%)	9 (13.2%)	
4	18 (22.0%)^+^	27 (39.7%)^+^	

Descriptive statistics were presented by using mean ± standard deviation for normally distributed continuous variables, median (minimum–maximum) for non-normally distributed continuous variables and frequency (percentage) for categorical variables. *: Data was available for 114 patients, +: Significantly different category, a: Independent samples *t*-test, b: Chi-square test, c: Mann–Whitney *U* test. SOX9: SRY-box transcription factor 9; TNM: Tumor-node-metastasis.

**Table 4:** Demographics and tumor characteristics of patients with regard to SOX9 proportion score

**SOX9 proportion score**			
**Variables**	**≤66% (*n* ═ 86)**	**>66% (*n* ═ 64)**	***P* value**
Age	67.1 ± 12.4	67.6 ± 11.2	0.799^a^
*Sex*			
Female	27 (31.4%)	17 (26.6%)	0.520^b^
Male	59 (68.6%)	47 (73.4%)	
*Differentiation*			
Well	6 (7.0%)	2 (3.1%)	
Moderate	39 (45.3%)	31 (48.4%)	0.577^b^
Poor	41 (47.7%)	31 (48.4%)	
Tumor size, cm	5 (1–15)	5 (1–15)	0.057^c^
*Subtype*			
Tubular	49 (57.0%)	36 (56.3%)	
Papillary	7 (8.1%)	6 (9.4%)	0.994^b^
Mucinous	10 (11.6%)	7 (10.9%)	
Poorly cohesive	20 (23.3%)	15 (23.4%)	
Vascular invasion	47 (54.7%)	44 (68.8%)	0.080^b^
Perineural invasion	39 (45.3%)	43 (67.2%)	0.008^b^
Lymph node metastasis	60 (69.8%)	49 (76.6%)	0.356^b^
Distance metastasis*	22 (32.8%)	23 (48.9%)	0.083^b^
*T staging*			
T1	11 (12.8%)	4 (6.3%)	
T2	11 (12.8%)	3 (4.7%)	0.031^b^
T3	30 (34.9%)	17 (26.6%)	
T4	34 (39.5%)^+^	40 (62.5%)^+^	
*Lymph node staging*			
N0	26 (30.2%)	15 (23.4%)	
N1	14 (16.3%)	7 (10.9%)	
N2	14 (16.3%)	7 (10.9%)	0.261^b^
N3a	18 (20.9%)	23 (35.9%)	
N3b	14 (16.3%)	12 (18.8%)	
*Total staging*			
1A	8 (9.3%)	3 (4.7%)	
1B	7 (8.1%)	3 (4.7%)	
2A	12 (14.0%)	7 (10.9%)	
2B	5 (5.8%)	2 (3.1%)	0.770^b^
3A	13 (15.1%)	10 (15.6%)	
3B	11 (12.8%)	10 (15.6%)	
3C	8 (9.3%)	6 (9.4%)	
4	22 (25.6%)	23 (35.9%)	

Descriptive statistics were presented by using mean ± standard deviation for normally distributed continuous variables, median (minimum–maximum) for non-normally distributed continuous variables and frequency (percentage) for categorical variables. *: Data was available for 114 patients, +: Significantly different category, a: Independent samples *t*-test, b: Chi-square test, c: Mann–Whitney *U* test. SOX9: SRY-box transcription factor 9.

In the regression analyses, the total SOX immune staining score emerged as a consistent predictor for vascular invasion (β ═ 0.790, *P* ═ 0.0257), perineural invasion (β ═ 0.972, *P* ═ 0.0045), and TNM stage (β ═ −1.178, *P* ═ 0.0003). In contrast, the SOX9 proportion score was significantly associated only with lymph node metastasis (β ═ 2.709, *P* ═ 0.0349). The SOX9 intensity score demonstrated strong links with vascular invasion at levels 1–3 (β ═ −1.665, *P* ═ 0.0032) and 2–3 (β ═ −0.954, *P* ═ 0.0161), while showing borderline significance for perineural invasion (β ═ −0.931, *P* ═ 0.0791) and distant metastasis (β ═ −2.235, *P* ═ 0.0795). These findings partially align with earlier studies that highlighted SOX9’s role in influencing tumor size, differentiation, and metastasis. However, the regression analyses revealed fewer significant associations with outcomes like tumor size and differentiation.

## Discussion

The primary objective of this study was to elucidate the relationship between SOX9 expression and the pathological and clinical characteristics of gastric adenocarcinomas, as well as its predictive significance in the progression and prognosis of GC. This investigation, conducted on a Turkish cohort of 150 patients with clinicopathological data, revealed moderate to strong SOX9 intensity in 85.3% of cases, with high SOX9 expression observed in 40% of gastric carcinoma cases.

Our findings demonstrate that patients with elevated SOX9 expression or intensity tend to exhibit higher T and lymph node staging. We identified a positive correlation between SOX9 expression and intensity scores and several key prognostic factors, including tumor size and differentiation, vascular and perineural invasion, and the presence of lymph node and distant metastasis. Notably, our study highlighted a progressive deterioration in prognostic factors as SOX9 expression or intensity scores increased. Collectively, these findings align with the majority of the literature in suggesting that SOX9 may play a pivotal role in shaping the clinicopathological profile of gastric adenocarcinomas. Furthermore, SOX9 could serve as a valuable predictive marker for the progression and prognosis of GC.

Regression analyses underscored the importance of the SOX9 immune staining score, which emerged as a consistent predictor of TNM stage, vascular invasion, and perineural invasion. By contrast, the SOX9 proportion score was significantly associated only with lymph node metastasis, suggesting that the total immune staining score may provide a more comprehensive measure of SOX9’s overall impact. Additionally, the SOX9 intensity score demonstrated significant associations with vascular invasion across multiple levels, as well as borderline significance for perineural invasion and distant metastasis, further supporting its utility in understanding SOX9’s role in GC progression.

The molecular pathogenesis of GC is exceedingly complex, involving numerous genetic and epigenetic alterations that dysregulate multiple pathways. While the precise mechanisms underlying disease onset and progression remain unclear [21], it is evident that the pathophysiology is influenced by a myriad of factors rather than a single dysfunction. Established evidence indicates that age, sex, presenting symptoms, clinical stage, pathological TNM stage, depth of tumor invasion, tumor size and location, tumor histological type, and the presence of lymph node and distant metastasis significantly affect GC prognosis [22].

SOX9 is a multifaceted regulator with direct and indirect effects on cellular functions, particularly within the intestinal tract. Key physiological processes associated with SOX9 include proliferation, cell fate determination, cell maintenance, apoptosis, invasion, and tumorigenesis [23]. SOX9 is expressed in both healthy and cancerous epithelium, and its expression is regulated by the Wnt pathway. Inactivation of the *SOX9* gene in intestinal epithelium leads to epithelial hyperplasia and focal crypt dysplasia, underscoring its regulatory role in cell proliferation [24]. SOX9 also promotes uncontrolled proliferation and malignant properties in tumor cells by inhibiting *INK4A/ARF* expression through *BMI-1* induction [25]. While the overexpression of SOX9 has been widely documented in (pre)cancerous gastrointestinal lesions, stronger evidence exists for its role in non-gastric tissues. Nevertheless, its correlation with tumor aggressiveness highlights SOX9 as a compelling, though complex, target for the development of more potent cancer therapies [13].

The past few years have seen a surge in studies examining the role of SOX9 in GC, but their results remain contradictory, as evidenced by data obtained from meta-analyses [13, 14]. A detailed analysis of several notable studies can help clarify the disparities in findings. For instance, Kimura et al. [26] demonstrated in clinical samples that SOX9 is expressed in intestinal metaplasia and gastric carcinoma tissues. Mezquita et al. [17], in a study of 333 cases, reported that SOX9 expression was not associated with clinicopathological features but was linked to a lower risk of relapse in patients with GC. Similarly, Zhang et al. [27] observed that SOX9 expression was significantly higher in GC tissues compared to adjacent normal tissues, though it was not associated with lymph node and distant metastasis, TNM staging, or tumor size. In a study involving 185 patients with gastric carcinoma, Choi et al. [28] reported no significant associations between SOX9 protein expression and clinical or pathological characteristics, including tumor invasion, lymph node metastasis, poor differentiation, or overall survival. These findings contrast with a meta-analysis of 11 articles involving 3060 GC patients, which reported that SOX9 expression was associated with TNM staging, tumor invasion depth, and poor overall survival, but not with age, sex, differentiation, or lymph node metastasis [14]. Additionally, a retrospective study by Shao et al. [29] involving 112 patients found significant relationships between SOX9 expression and tumor invasion stage, lymph node stage, and distant metastasis—components of the TNM stage. Santos et al. also reported significant correlations between SOX9 expression and TNM stage [30], further supporting the majority of literature while highlighting the heterogeneity in findings across meta-analyses. Given these conflicting results, we sought to examine the components of the TNM stage separately and take an overall approach. While we found a significant relationship between SOX9 staining intensity and TNM stage, no significant correlation was observed between SOX9 staining proportion and TNM stage. Additionally, our findings revealed that SOX9 staining intensity and expression increased as the TNM stage advanced. These results suggest that SOX9 staining intensity may serve as an indicator of increased tumor malignancy and disease progression, potentially making it a prognostic marker for GC.

We also investigated the impact of SOX9 expression and intensity scores on the clinical and pathological characteristics of GC patients. Our analysis showed that positive SOX9 expression and intensity scores were significantly associated with tumor size, differentiation, vascular and perineural invasion, and the presence of lymph node and distant metastases. Consistent with the majority of the literature, no significant correlations were observed with age, sex, or histological subtype. Specifically, patients with vascular or perineural invasion, poor tumor differentiation, larger tumor size, or lymph node metastasis exhibited markedly higher SOX9 intensity and expression scores. Interestingly, our results diverged from those reported by Sun et al., who found an inverse correlation between SOX9 expression and vascular invasion, advanced tumor stage, and nodal metastasis [31]. Mezquita et al. [17] also reported no relationship between SOX9 expression and perineural invasion, while suggesting no association between SOX9 and WHO classification. Conversely, our findings align with those of Link et al. [12], who demonstrated a significant relationship between SOX9 expression and lymph node metastasis in 199 cases. Additionally, Wang et al. [13], in a meta-analysis of 17 studies comprising 2893 GC patients, reported that SOX9 expression was associated with age, tumor size, histological differentiation, tumor stage, lymph node metastasis, advanced TNM staging, and poor overall survival, but not with sex, vascular invasion, or distant metastasis. These variations in findings, both between studies and among meta-analyses, underscore the complex interaction between SOX9 and GC characteristics, particularly regarding in vivo behavior. Our results indicate that SOX9 is closely associated with GC occurrence and progression. SOX9 may hold potential as a target for the diagnosis and management of GC. However, despite advances in understanding mechanisms involving cellular pathways, T cell activity, tumor microenvironment, inflammation, oxidative stress, and their collective influence on tissue behavior [32–36], further studies with advanced methodologies are necessary to address the complexities of human disease. The observed discrepancies between studies may stem from the multifaceted roles of SOX9 in GC, influenced by factors, such as population characteristics, tumor stage, degrees of differentiation, and potential expression-level-dependent effects. Additionally, variations in cutoff values used to define SOX9 subgroups likely contribute to the inconsistencies.

Since no universally accepted scoring system exists for assessing SOX9 antibody expression, some studies prioritize nuclear staining proportion, while others consider intensity scores to be more predictive [27, 31, 37]. To enhance the discriminative power of the SOX9 proportion score, we applied cutoff values of 0%, 1%, 10%, 33%, and 66%, calculated based on the ratio of positively stained tumor cells to the total tumor cell count, as recommended by Yuan et al. [20]. We also calculated an expression score by multiplying the proportion and intensity scores, enabling us to explore the independent association of these three scores with prognostic factors. To our knowledge, this is the first study to evaluate both the proportion and intensity of SOX9 expression in gastric carcinomas. Future research should focus on standardizing methodologies for SOX9 evaluation to facilitate comparisons across studies and improve our understanding of its role in GC.

The study is subject to several limitations that should be acknowledged. First, the retrospective design inherently introduces biases due to the reliance on historical data. Additionally, the single-center data collection may have led to detection and ascertainment bias. Second, while the sample size included a substantial number of patients, it may still have been insufficient to identify subtle relationships in subgroup analyses. Furthermore, some data points were limited, such as missing information on distant organ metastasis for 36 patients, which raises concerns about analyses involving prognostic and outcome-related relationships. This missing data could reflect selection bias, as it may disproportionately affect patients with similar characteristics or disease severity, potentially leading to an incomplete representation of patients with GC. That said, as a tertiary healthcare institution, our center likely mitigates some risks of selection bias and underrepresentation. Another limitation is the lack of accessible survival data, which prevented a thorough prognostic evaluation. In conclusion, while this study provides valuable insights into the relationship between SOX9 expression and GC, addressing these limitations will be essential in future research. Larger, multicenter cohorts and prospective study designs are recommended to validate and expand upon these findings.

## Conclusion

We demonstrate that SOX9 may influence critical factors, such as tumor size, differentiation, vascular and perineural invasion, TNM staging, and lymph node or distant metastases in GC. However, no associations were observed with age, sex, or histological subtype. The increased expression of SOX9 protein and higher intensity scores in GC appear to correlate with an unfavorable prognosis. To strengthen and validate these findings, additional prospective, multicenter studies with larger sample sizes are needed. Such investigations would enhance the robustness of our current data, particularly in establishing SOX9 as a potential pathological prognostic indicator for GC.
